# 9-[(2,6-Dimethoxy­phen­oxy)carbon­yl]-10-methyl­acridinium trifluoro­methane­sulfonate

**DOI:** 10.1107/S1600536809007570

**Published:** 2009-03-19

**Authors:** Karol Krzymiński, Damian Trzybiński, Artur Sikorski, Jerzy Błażejowski

**Affiliations:** a Faculty of Chemistry, University of Gdańsk, J. Sobieskiego 18, 80-952 Gdańsk, Poland

## Abstract

In the crystal structure of the title compound, C_23_H_20_NO_4_^+^·CF_3_SO_3_^−^, the cations are linked through C—H⋯O, C—H⋯π and π–π inter­actions [centroid-centroid distances = 3.641 (2) and 3.885 (2) Å]. The cation and the anion are held together by C—H⋯O and S—O⋯π inter­actions. The acridine ring system and the benzene ring in the cation are oriented at a dihedral angle of 8.7 (1)°. The carb­oxy group is twisted at an angle of 83.2 (1)° relative to the acridine skeleton.

## Related literature

For general background, see: Adamczyk *et al.* (2004 ▶); Becker *et al.* (1999 ▶); Rak *et al.* (1999 ▶); Zomer & Jacquemijns (2001 ▶). For related structures, see: Sikorski *et al.* (2008 ▶). For mol­ecular inter­actions, see: Bianchi *et al.* (2004 ▶); Dorn *et al.* (2005 ▶); Hunter *et al.* (2001 ▶); Steiner (1999 ▶); Takahashi *et al.* (2001 ▶). For the synthesis, see: Sato (1996 ▶).
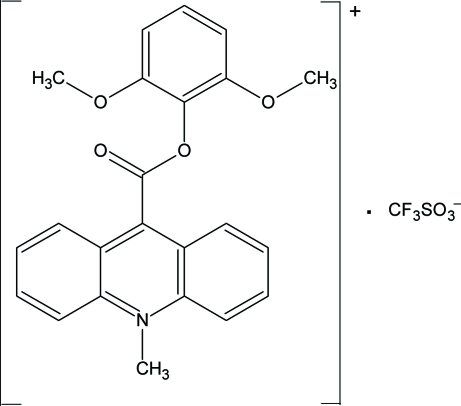


## Experimental

### 

#### Crystal data

C_23_H_20_NO_4_^+^·CF_3_SO_3_^−^
*M* *_r_* = 523.48
Monoclinic, 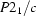

*a* = 11.6803 (4) Å
*b* = 14.7434 (5) Å
*c* = 13.6286 (5) Å
β = 93.462 (4)°
*V* = 2342.66 (14) Å^3^
*Z* = 4
Mo *K*α radiation
μ = 0.21 mm^−1^
*T* = 295 K
0.55 × 0.30 × 0.02 mm

#### Data collection

Oxford Diffraction Gemini R Ultra Ruby CCD diffractometer
Absorption correction: multi-scan (**CrysAlis RED**; Oxford Diffraction, 2008 ▶) *T*_min_ = 0.911, *T*_max_ = 0.995
20680 measured reflections
4160 independent reflections
2274 reflections with *I* > 2σ(*I*)
*R*_int_ = 0.045

#### Refinement

*R*[*F* ^2^ > 2σ(*F* ^2^)] = 0.039
*wR*(*F* ^2^) = 0.109
*S* = 0.87
4160 reflections
328 parameters
H-atom parameters constrained
Δρ_max_ = 0.24 e Å^−3^
Δρ_min_ = −0.29 e Å^−3^

### 

Data collection: *CrysAlis CCD* (Oxford Diffraction, 2008 ▶); cell refinement: *CrysAlis RED* (Oxford Diffraction, 2008 ▶); data reduction: *CrysAlis RED*; program(s) used to solve structure: *SHELXS97* (Sheldrick, 2008 ▶); program(s) used to refine structure: *SHELXL97* (Sheldrick, 2008 ▶); molecular graphics: *ORTEP-3* (Farrugia, 1997 ▶); software used to prepare material for publication: *SHELXL97* and *PLATON* (Spek, 2009 ▶).

## Supplementary Material

Crystal structure: contains datablocks global, I. DOI: 10.1107/S1600536809007570/is2390sup1.cif

Structure factors: contains datablocks I. DOI: 10.1107/S1600536809007570/is2390Isup2.hkl

Additional supplementary materials:  crystallographic information; 3D view; checkCIF report

## Figures and Tables

**Table 1:** Hydrogen-bond geometry (Å, °)

*D*—H⋯*A*	*D*—H	H⋯*A*	*D*⋯*A*	*D*—H⋯*A*
C3—H3⋯O30^i^	0.93	2.57	3.449 (3)	158
C4—H4⋯O31^i^	0.93	2.58	3.352 (3)	141
C7—H7⋯O32^ii^	0.93	2.54	3.427 (3)	159
C27—H27*C*⋯O17^iii^	0.96	2.46	3.371 (3)	159
C25—H25*C*⋯*Cg*4^iv^	0.96	2.98	3.845 (3)	150

Symmetry codes: (i) 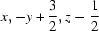; (ii) 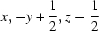; (iii) 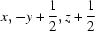; (iv) 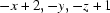. *Cg*4 is the centroid of the C18–C23 ring.

**Table 2:** S—O⋯π Interactions (Å,°)

*X*	*I*	*J*	*I*⋯*J*	*X*⋯*J*	*X*—*I*⋯*J*
S29	O32	*Cg*1^v^	3.178 (2)	3.757 (2)	103

Symmetry codes: (v) –*x*+1, –*y*+1, –*z*+1. *Cg*1 is the centroid of the C9/N10/C11–C14 ring.

**Table 3:** π–π Interactions (Å,°)

*I*	*J*	*CgI*⋯*CgJ*	Dihedral angle	*CgI*_Perp_	*CgJ*_Perp_	*CgI*_Offset_	*CgJ*_Offset_
1	4^ii^	3.641 (2)	5.31 (10)	3.416 (2)	3.492 (2)	0.767 (2)	1.031 (2)
2	4^ii^	3.885 (2)	6.74 (11)	3.666 (2)	3.491 (2)	1.286 (2)	1.705 (2)

Symmetry code: (ii) 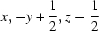. Notes: *Cg*1, *Cg*2 and *Cg*4 are the centroids of the C9/N10/C11–C14, C1–C4/C11/C12 and C18–C23 rings, respectively. *CgI*⋯*CgJ* is the distance between ring centroids. The dihedral angle is that between the planes of the rings *I* and *J*. *CgI*_Perp_ and *CgJ*_Perp_ are the perpendicular distances of *CgI* from ring *J* and of *CgJ* from ring *I*, respectively. *CgI*_Offset_ and *CgJ*_Offset_ are the distances between *CgI* and the perpendicular projection of *CgJ* on ring *I*, and between *CgJ* and the perpendicular projection of *CgI* on ring *J*, respectively.

**Fig. 1.**
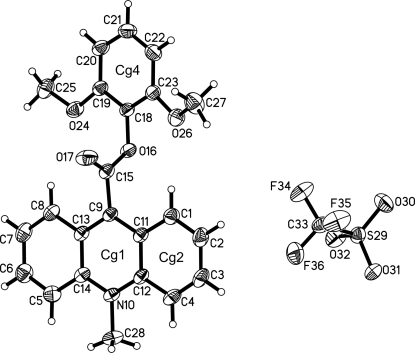
The molecular structure of the title compound showing the atom-labeling scheme. Displacement ellipsoids are drawn at the 25% probability level, and H atoms are shown as small spheres of arbitrary radius. Cg1, Cg2 and Cg4 denote the ring centroids.

**Fig. 2.**
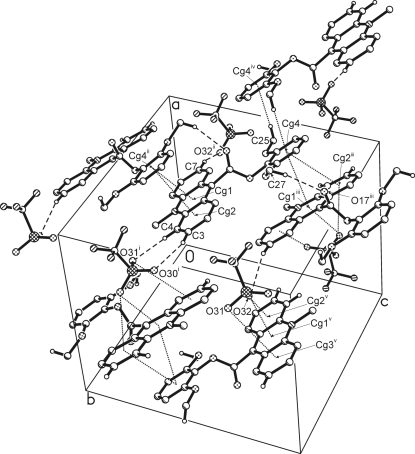
The arrangement of the ions in the crystal structure, the C—H···O interactions are represented by dashed lines, the C—H···π, S—O···π and π–π contacts by dotted lines. H atoms not involved in interactions have been omitted. [Symmetry codes: (i) x, -y + 3/2, z - 1/2; (ii) x, -y + 1/2, z - 1/2; (iii) x, -y + 1/2, z + 1/2; (iv) -x + 2, -y, -z + 1; (v) -x + 1, -y + 1, -z + 1.]

## supplementary crystallographic information

### Comment

Phenyl 10-alkylacridinium-9-carboxylates have long been known as chemiluminescent indicators or the chemiluminogenic fragments of chemiluminescent labels (Zomer & Jacquemijns, 2001). These compounds are widely applied in assays of biologically and environmentally important entities such as antigens, antibodies, enzymes or DNA fragments (Becker *et al.*, 1999; Adamczyk *et al.*, 2004). The reaction of the cations of these salts with hydrogen peroxide in alkaline media produces light. Our own investigations (Rak *et al.*, 1999) and those of others (Zomer & Jacquemijns, 2001) have revealed that oxidation of acridinium chemiluminogens is accompanied by the removal of the phenoxycarbonyl fragment and the convertion of the rest of molecules to electronically excited, light-emitting 10-alkyl-9-acridinones. It has been found that the efficiency of chemiluminescence is affected by the constitution of the phenyl fragment (Zomer & Jacquemijns, 2001). Continuing our investigations onto the above mentioned effect, we synthesized the compound containing two methoxy groups in the phenyl fragment. Here, we present its structure. Methoxy groups, which possess electron-attractive features, may influence the stability and chemiluminogenic ability of the compound investigated.

In the cation of the title compound (Fig. 1), the bond lengths and angles characterizing the geometry of the acridinium moiety are typical of acridine-based derivatives (Sikorski *et al.*, 2008). With respective average deviations from planarity of 0.037 (3) Å and 0.010 (3) Å, the acridine and benzene ring systems in the cation are oriented at 8.7 (1)°. The carboxy group is twisted at an angle of 83.2 (1)° relative to the acridine skeleton. The mean planes of the adjacent acridine moieties are either parallel or inclined at an angle of 10.9 (1)° in the lattice.

In the crystal structure, the cations are linked through C—H···O (Table 1, Fig. 2), C—H···π (Table 1, Fig. 2) and π–π (Table 3, Fig. 2) interactions, and the cations and anions by C—H···O (Table 1, Fig. 2) and S—O···π (Table 2, Fig. 2) interactions. The C—H···O (Steiner, 1999; Bianchi *et al.*, 2004) interactions are of the hydrogen-bond type. The C—H···π (Takahashi *et al.*, 2001) and S—O···π (Dorn *et al.*, 2005) interactions should be of an attractive nature, like the π–π contacts (Hunter *et al.*, 2001). The crystal structure is stabilized by a network of the aforementioned short-range specific interactions and by long-range electrostatic interactions between ions.

### Experimental

2,6-Dimethoxyphenylacridine-9-carboxylate was prepared by heating anhydrous acridine-9-carboxylic acid with thionyl chloride, followed by esterification of the resulting acid chloride with an equimolar quantity of 2,6-dimethoxyphenol (Sato, 1996). The reaction was carried out in anhydrous dichloromethane in the presence of *N*,*N*-diethylethanamine (1.5 molar excess) and a catalytic amount of *N*,*N*-dimethyl-4-pyridinamine (room temperature, 15 - 25 h). The crude product was purified chromatographically (SiO_2_, cyclohexane/ethyl acetate, 3/2 *v*/*v*). The 2,6-dimethoxyphenylacridine-9-carboxylate thus obtained was subsequently dissolved in anhydrous dichloromethane and treated with a fivefold molar excess of methyl triluoromethanesulfonate dissolved in the same solvent (under an Ar atmosphere at room temperature for 4 h). The crude salt was dissolved in a small amount of ethanol, filtered and precipitated with a 25 *v*/*v* excess of diethyl ether (yield 42%). Yellow crystals suitable for X-ray investigations were grown from absolute ethanol solution (m.p. 243–245 K).

### Refinement

H atoms were positioned geometrically, with C—H = 0.93 and 0.96 Å for the aromatic and methyl H atoms, respectively, and constrained to ride on their parrent atoms with *U*_iso_(H) = *xU*_eq_(C), where *x* = 1.2 for the aromatic and *x* = 1.5 for the methyl H atoms.

### Crystal data

**Table:**

C_23_H_20_NO_4_^+^·CF_3_SO_3_^−^	*F*(000) = 1080
*M**_r_* = 523.48	*D*_x_ = 1.484 Mg m^−^^3^
Monoclinic, *P*2_1_/*c*	Mo *K*α radiation, λ = 0.71073 Å
Hall symbol: -p 2ybc	Cell parameters from 6747 reflections
*a* = 11.6803 (4) Å	θ = 3.1–29.2°
*b* = 14.7434 (5) Å	µ = 0.21 mm^−^^1^
*c* = 13.6286 (5) Å	*T* = 295 K
β = 93.462 (4)°	Plate, yellow
*V* = 2342.66 (14) Å^3^	0.55 × 0.30 × 0.02 mm
*Z* = 4	

### Data collection

**Table:**

Oxford Diffraction Gemini R Ultra Ruby CCD diffractometer	4160 independent reflections
Radiation source: Enhanced (Mo) X-ray Source	2274 reflections with *I* > 2σ(*I*)
graphite	*R*_int_ = 0.045
Detector resolution: 10.4002 pixels mm^-1^	θ_max_ = 25.1°, θ_min_ = 3.1°
ω scans	*h* = −13→13
Absorption correction: multi-scan (*CrysAlis RED*; Oxford Diffraction, 2008)	*k* = −17→17
*T*_min_ = 0.911, *T*_max_ = 0.995	*l* = −15→16
20680 measured reflections	

### Refinement

**Table:**

Refinement on *F*^2^	Primary atom site location: structure-invariant direct methods
Least-squares matrix: full	Secondary atom site location: difference Fourier map
*R*[*F*^2^ > 2σ(*F*^2^)] = 0.039	Hydrogen site location: inferred from neighbouring sites
*wR*(*F*^2^) = 0.109	H-atom parameters constrained
*S* = 0.87	*w* = 1/[σ^2^(*F*_o_^2^) + (0.0672*P*)^2^] where *P* = (*F*_o_^2^ + 2*F*_c_^2^)/3
4160 reflections	(Δ/σ)_max_ = 0.001
328 parameters	Δρ_max_ = 0.24 e Å^−^^3^
0 restraints	Δρ_min_ = −0.29 e Å^−^^3^

### Special details

**Table:**

Geometry. All e.s.d.'s (except the e.s.d. in the dihedral angle between two l.s. planes) are estimated using the full covariance matrix. The cell e.s.d.'s are taken into account individually in the estimation of e.s.d.'s in distances, angles and torsion angles; correlations between e.s.d.'s in cell parameters are only used when they are defined by crystal symmetry. An approximate (isotropic) treatment of cell e.s.d.'s is used for estimating e.s.d.'s involving l.s. planes.

### Fractional atomic coordinates and isotropic or equivalent isotropic displacement parameters (Å^2^)

**Table:**

	*x*	*y*	*z*	*U*_iso_*/*U*_eq_	
C1	0.7814 (2)	0.40388 (17)	0.39746 (18)	0.0638 (6)	
H1	0.8256	0.3737	0.4462	0.077*	
C2	0.7685 (2)	0.49461 (18)	0.4029 (2)	0.0758 (8)	
H2	0.8031	0.5265	0.4556	0.091*	
C3	0.7034 (2)	0.54073 (18)	0.3296 (2)	0.0732 (8)	
H3	0.6953	0.6033	0.3341	0.088*	
C4	0.6517 (2)	0.49643 (17)	0.2521 (2)	0.0651 (7)	
H4	0.6096	0.5289	0.2038	0.078*	
C5	0.5619 (2)	0.2128 (2)	0.08150 (18)	0.0665 (7)	
H5	0.5216	0.2437	0.0310	0.080*	
C6	0.5684 (2)	0.1217 (2)	0.0792 (2)	0.0759 (8)	
H6	0.5322	0.0908	0.0265	0.091*	
C7	0.6277 (2)	0.07213 (19)	0.1532 (2)	0.0748 (7)	
H7	0.6284	0.0091	0.1505	0.090*	
C8	0.6843 (2)	0.11584 (17)	0.22920 (18)	0.0622 (6)	
H8	0.7255	0.0828	0.2776	0.075*	
C9	0.73852 (17)	0.26032 (15)	0.31071 (15)	0.0471 (6)	
N10	0.61009 (14)	0.35392 (13)	0.16713 (13)	0.0508 (5)	
C11	0.72823 (17)	0.35398 (15)	0.31827 (15)	0.0497 (6)	
C12	0.66127 (18)	0.40128 (15)	0.24392 (16)	0.0503 (6)	
C13	0.68078 (18)	0.21248 (15)	0.23483 (16)	0.0495 (6)	
C14	0.61625 (18)	0.26140 (16)	0.16052 (16)	0.0514 (6)	
C15	0.8199 (2)	0.21059 (14)	0.38169 (16)	0.0523 (6)	
O16	0.77183 (12)	0.18961 (10)	0.46502 (11)	0.0556 (4)	
O17	0.91548 (15)	0.19252 (14)	0.36460 (13)	0.0862 (6)	
C18	0.84322 (18)	0.14411 (16)	0.53585 (15)	0.0512 (6)	
C19	0.84232 (19)	0.05032 (16)	0.53766 (17)	0.0548 (6)	
C20	0.9070 (2)	0.00594 (18)	0.61157 (19)	0.0657 (7)	
H20	0.9073	−0.0570	0.6151	0.079*	
C21	0.9708 (2)	0.0567 (2)	0.67943 (19)	0.0742 (8)	
H21	1.0134	0.0270	0.7296	0.089*	
C22	0.9739 (2)	0.1498 (2)	0.67584 (17)	0.0700 (7)	
H22	1.0185	0.1823	0.7224	0.084*	
C23	0.9101 (2)	0.19452 (17)	0.60234 (16)	0.0569 (6)	
O24	0.77711 (14)	0.01014 (11)	0.46366 (12)	0.0674 (5)	
C25	0.7774 (2)	−0.08702 (17)	0.4597 (2)	0.0771 (8)	
H25A	0.7387	−0.1068	0.3993	0.116*	
H25B	0.7386	−0.1108	0.5143	0.116*	
H25C	0.8551	−0.1086	0.4629	0.116*	
O26	0.90647 (16)	0.28607 (12)	0.58923 (12)	0.0754 (5)	
C27	0.9977 (3)	0.3383 (2)	0.6341 (2)	0.0978 (10)	
H27A	0.9918	0.3998	0.6111	0.147*	
H27B	1.0698	0.3132	0.6172	0.147*	
H27C	0.9932	0.3372	0.7042	0.147*	
C28	0.5417 (2)	0.40504 (19)	0.09069 (19)	0.0798 (8)	
H28A	0.5904	0.4472	0.0593	0.120*	
H28B	0.4816	0.4376	0.1205	0.120*	
H28C	0.5086	0.3635	0.0426	0.120*	
S29	0.62429 (6)	0.72700 (4)	0.67537 (5)	0.0605 (2)	
O30	0.6575 (2)	0.73286 (15)	0.77722 (13)	0.1141 (8)	
O31	0.59056 (15)	0.81084 (11)	0.62945 (13)	0.0719 (5)	
O32	0.55268 (15)	0.65261 (13)	0.64848 (15)	0.0913 (6)	
C33	0.7559 (2)	0.70003 (18)	0.6202 (2)	0.0687 (7)	
F34	0.79921 (14)	0.62064 (11)	0.65070 (14)	0.1050 (6)	
F35	0.83549 (13)	0.76166 (12)	0.64108 (16)	0.1158 (6)	
F36	0.74067 (16)	0.69624 (13)	0.52297 (12)	0.1107 (6)	

### Atomic displacement parameters (Å^2^)

**Table:**

	*U*^11^	*U*^22^	*U*^33^	*U*^12^	*U*^13^	*U*^23^
C1	0.0710 (16)	0.0497 (17)	0.0707 (15)	−0.0017 (13)	0.0038 (13)	0.0012 (13)
C2	0.095 (2)	0.0490 (18)	0.0841 (19)	−0.0063 (15)	0.0125 (16)	−0.0089 (14)
C3	0.0845 (19)	0.0378 (15)	0.099 (2)	0.0033 (14)	0.0219 (17)	0.0021 (16)
C4	0.0616 (16)	0.0456 (17)	0.0894 (19)	0.0057 (12)	0.0156 (14)	0.0186 (14)
C5	0.0560 (15)	0.071 (2)	0.0721 (17)	−0.0045 (14)	−0.0009 (12)	0.0024 (15)
C6	0.0752 (18)	0.065 (2)	0.0870 (19)	−0.0106 (15)	−0.0002 (15)	−0.0110 (16)
C7	0.0789 (18)	0.0448 (16)	0.101 (2)	−0.0049 (14)	0.0100 (16)	−0.0090 (16)
C8	0.0645 (15)	0.0417 (16)	0.0806 (17)	0.0021 (12)	0.0055 (13)	0.0037 (13)
C9	0.0448 (12)	0.0396 (14)	0.0578 (13)	0.0018 (10)	0.0112 (11)	0.0103 (11)
N10	0.0406 (10)	0.0473 (13)	0.0649 (12)	0.0041 (9)	0.0070 (9)	0.0127 (10)
C11	0.0483 (13)	0.0400 (14)	0.0616 (14)	−0.0005 (11)	0.0109 (11)	0.0071 (12)
C12	0.0464 (13)	0.0385 (15)	0.0675 (15)	0.0019 (11)	0.0170 (12)	0.0087 (12)
C13	0.0456 (12)	0.0412 (15)	0.0628 (14)	−0.0006 (11)	0.0114 (11)	0.0057 (11)
C14	0.0410 (12)	0.0483 (15)	0.0658 (15)	0.0006 (11)	0.0104 (11)	0.0082 (12)
C15	0.0512 (15)	0.0434 (15)	0.0634 (15)	0.0005 (11)	0.0114 (12)	0.0072 (11)
O16	0.0543 (9)	0.0517 (10)	0.0617 (9)	0.0094 (7)	0.0104 (8)	0.0111 (8)
O17	0.0573 (11)	0.1193 (17)	0.0841 (12)	0.0260 (10)	0.0210 (9)	0.0410 (11)
C18	0.0500 (13)	0.0538 (16)	0.0505 (13)	0.0100 (12)	0.0085 (11)	0.0076 (12)
C19	0.0551 (14)	0.0506 (16)	0.0596 (15)	0.0053 (12)	0.0115 (12)	0.0081 (13)
C20	0.0700 (16)	0.0562 (17)	0.0721 (17)	0.0092 (14)	0.0144 (14)	0.0178 (14)
C21	0.0784 (19)	0.081 (2)	0.0628 (16)	0.0142 (16)	0.0023 (14)	0.0194 (16)
C22	0.0726 (17)	0.081 (2)	0.0564 (15)	0.0022 (15)	0.0027 (13)	−0.0005 (14)
C23	0.0622 (15)	0.0554 (17)	0.0542 (14)	0.0075 (13)	0.0131 (13)	0.0059 (13)
O24	0.0732 (11)	0.0490 (11)	0.0794 (11)	−0.0003 (9)	−0.0007 (9)	0.0070 (9)
C25	0.0817 (19)	0.0502 (18)	0.1008 (19)	−0.0032 (14)	0.0178 (15)	−0.0019 (15)
O26	0.0934 (13)	0.0544 (12)	0.0776 (11)	−0.0019 (10)	−0.0010 (10)	−0.0035 (9)
C27	0.126 (3)	0.083 (2)	0.0849 (19)	−0.028 (2)	0.0082 (18)	−0.0061 (17)
C28	0.0725 (18)	0.074 (2)	0.0911 (18)	0.0139 (15)	−0.0125 (14)	0.0230 (16)
S29	0.0693 (4)	0.0491 (4)	0.0639 (4)	−0.0018 (3)	0.0105 (3)	0.0018 (3)
O30	0.183 (2)	0.1048 (17)	0.0545 (11)	0.0155 (16)	0.0050 (12)	0.0030 (11)
O31	0.0811 (12)	0.0492 (11)	0.0853 (11)	0.0124 (9)	0.0036 (9)	0.0049 (9)
O32	0.0713 (12)	0.0602 (13)	0.1427 (16)	−0.0205 (10)	0.0087 (11)	0.0027 (12)
C33	0.0625 (17)	0.0533 (17)	0.089 (2)	−0.0042 (14)	−0.0076 (14)	0.0083 (14)
F34	0.0808 (11)	0.0678 (11)	0.1654 (16)	0.0192 (9)	−0.0016 (10)	0.0175 (11)
F35	0.0617 (10)	0.0977 (13)	0.1853 (18)	−0.0272 (10)	−0.0152 (10)	0.0345 (12)
F36	0.1302 (15)	0.1217 (16)	0.0842 (12)	0.0248 (11)	0.0384 (11)	−0.0103 (10)

### Geometric parameters (Å, °)

**Table:**

C1—C2	1.349 (3)	C18—C23	1.377 (3)
C1—C11	1.418 (3)	C18—C19	1.383 (3)
C1—H1	0.9300	C19—O24	1.362 (3)
C2—C3	1.396 (4)	C19—C20	1.387 (3)
C2—H2	0.9300	C20—C21	1.374 (3)
C3—C4	1.353 (3)	C20—H20	0.9300
C3—H3	0.9300	C21—C22	1.374 (4)
C4—C12	1.412 (3)	C21—H21	0.9300
C4—H4	0.9300	C22—C23	1.380 (3)
C5—C6	1.345 (4)	C22—H22	0.9300
C5—C14	1.412 (3)	C23—O26	1.362 (3)
C5—H5	0.9300	O24—C25	1.433 (3)
C6—C7	1.396 (4)	C25—H25A	0.9600
C6—H6	0.9300	C25—H25B	0.9600
C7—C8	1.357 (3)	C25—H25C	0.9600
C7—H7	0.9300	O26—C27	1.423 (3)
C8—C13	1.428 (3)	C27—H27A	0.9600
C8—H8	0.9300	C27—H27B	0.9600
C9—C11	1.390 (3)	C27—H27C	0.9600
C9—C13	1.392 (3)	C28—H28A	0.9600
C9—C15	1.506 (3)	C28—H28B	0.9600
N10—C12	1.366 (3)	C28—H28C	0.9600
N10—C14	1.369 (3)	S29—O32	1.4142 (19)
N10—C28	1.480 (3)	S29—O30	1.421 (2)
C11—C12	1.424 (3)	S29—O31	1.4300 (17)
C13—C14	1.422 (3)	S29—C33	1.796 (3)
C15—O17	1.184 (2)	C33—F35	1.319 (3)
C15—O16	1.334 (2)	C33—F36	1.327 (3)
O16—C18	1.407 (2)	C33—F34	1.332 (3)
			
C2—C1—C11	120.8 (2)	C19—C18—O16	118.9 (2)
C2—C1—H1	119.6	O24—C19—C18	115.2 (2)
C11—C1—H1	119.6	O24—C19—C20	126.1 (2)
C1—C2—C3	120.1 (3)	C18—C19—C20	118.7 (2)
C1—C2—H2	120.0	C21—C20—C19	118.8 (2)
C3—C2—H2	120.0	C21—C20—H20	120.6
C4—C3—C2	121.5 (2)	C19—C20—H20	120.6
C4—C3—H3	119.3	C20—C21—C22	122.3 (2)
C2—C3—H3	119.3	C20—C21—H21	118.8
C3—C4—C12	120.5 (2)	C22—C21—H21	118.8
C3—C4—H4	119.7	C21—C22—C23	119.3 (3)
C12—C4—H4	119.7	C21—C22—H22	120.4
C6—C5—C14	120.1 (2)	C23—C22—H22	120.4
C6—C5—H5	119.9	O26—C23—C18	115.9 (2)
C14—C5—H5	119.9	O26—C23—C22	125.5 (2)
C5—C6—C7	122.1 (3)	C18—C23—C22	118.7 (2)
C5—C6—H6	118.9	C19—O24—C25	117.39 (19)
C7—C6—H6	118.9	O24—C25—H25A	109.5
C8—C7—C6	120.0 (2)	O24—C25—H25B	109.5
C8—C7—H7	120.0	H25A—C25—H25B	109.5
C6—C7—H7	120.0	O24—C25—H25C	109.5
C7—C8—C13	120.0 (2)	H25A—C25—H25C	109.5
C7—C8—H8	120.0	H25B—C25—H25C	109.5
C13—C8—H8	120.0	C23—O26—C27	117.6 (2)
C11—C9—C13	121.2 (2)	O26—C27—H27A	109.5
C11—C9—C15	119.3 (2)	O26—C27—H27B	109.5
C13—C9—C15	119.3 (2)	H27A—C27—H27B	109.5
C12—N10—C14	122.52 (18)	O26—C27—H27C	109.5
C12—N10—C28	118.1 (2)	H27A—C27—H27C	109.5
C14—N10—C28	119.28 (19)	H27B—C27—H27C	109.5
C9—C11—C1	122.4 (2)	N10—C28—H28A	109.5
C9—C11—C12	118.7 (2)	N10—C28—H28B	109.5
C1—C11—C12	118.9 (2)	H28A—C28—H28B	109.5
N10—C12—C4	122.4 (2)	N10—C28—H28C	109.5
N10—C12—C11	119.4 (2)	H28A—C28—H28C	109.5
C4—C12—C11	118.2 (2)	H28B—C28—H28C	109.5
C9—C13—C14	119.0 (2)	O32—S29—O30	115.00 (13)
C9—C13—C8	122.1 (2)	O32—S29—O31	114.45 (12)
C14—C13—C8	118.9 (2)	O30—S29—O31	115.25 (12)
N10—C14—C5	122.2 (2)	O32—S29—C33	103.15 (12)
N10—C14—C13	119.1 (2)	O30—S29—C33	103.43 (14)
C5—C14—C13	118.7 (2)	O31—S29—C33	103.20 (11)
O17—C15—O16	124.5 (2)	F35—C33—F36	107.1 (2)
O17—C15—C9	123.3 (2)	F35—C33—F34	106.8 (2)
O16—C15—C9	112.20 (19)	F36—C33—F34	107.5 (2)
C15—O16—C18	115.60 (16)	F35—C33—S29	111.5 (2)
C23—C18—C19	122.2 (2)	F36—C33—S29	111.15 (18)
C23—C18—O16	118.9 (2)	F34—C33—S29	112.49 (19)
			
C11—C1—C2—C3	−0.7 (4)	C8—C13—C14—C5	−2.7 (3)
C1—C2—C3—C4	0.2 (4)	C11—C9—C15—O17	−94.4 (3)
C2—C3—C4—C12	0.8 (4)	C13—C9—C15—O17	81.4 (3)
C14—C5—C6—C7	0.1 (4)	C11—C9—C15—O16	85.7 (2)
C5—C6—C7—C8	−2.1 (4)	C13—C9—C15—O16	−98.5 (2)
C6—C7—C8—C13	1.7 (4)	O17—C15—O16—C18	1.0 (3)
C13—C9—C11—C1	176.74 (19)	C9—C15—O16—C18	−179.09 (18)
C15—C9—C11—C1	−7.6 (3)	C15—O16—C18—C23	88.2 (2)
C13—C9—C11—C12	−2.9 (3)	C15—O16—C18—C19	−93.1 (2)
C15—C9—C11—C12	172.79 (19)	C23—C18—C19—O24	−176.27 (18)
C2—C1—C11—C9	−179.3 (2)	O16—C18—C19—O24	5.0 (3)
C2—C1—C11—C12	0.3 (3)	C23—C18—C19—C20	2.9 (3)
C14—N10—C12—C4	−177.94 (19)	O16—C18—C19—C20	−175.80 (18)
C28—N10—C12—C4	−0.5 (3)	O24—C19—C20—C21	178.2 (2)
C14—N10—C12—C11	2.9 (3)	C18—C19—C20—C21	−0.9 (3)
C28—N10—C12—C11	−179.61 (19)	C19—C20—C21—C22	−0.9 (4)
C3—C4—C12—N10	179.7 (2)	C20—C21—C22—C23	0.7 (4)
C3—C4—C12—C11	−1.1 (3)	C19—C18—C23—O26	177.12 (18)
C9—C11—C12—N10	−0.6 (3)	O16—C18—C23—O26	−4.2 (3)
C1—C11—C12—N10	179.75 (18)	C19—C18—C23—C22	−3.1 (3)
C9—C11—C12—C4	−179.77 (18)	O16—C18—C23—C22	175.59 (18)
C1—C11—C12—C4	0.6 (3)	C21—C22—C23—O26	−179.0 (2)
C11—C9—C13—C14	4.1 (3)	C21—C22—C23—C18	1.3 (3)
C15—C9—C13—C14	−171.59 (19)	C18—C19—O24—C25	177.29 (18)
C11—C9—C13—C8	−175.94 (19)	C20—C19—O24—C25	−1.8 (3)
C15—C9—C13—C8	8.4 (3)	C18—C23—O26—C27	−160.7 (2)
C7—C8—C13—C9	−179.3 (2)	C22—C23—O26—C27	19.5 (3)
C7—C8—C13—C14	0.7 (3)	O32—S29—C33—F35	−177.69 (18)
C12—N10—C14—C5	179.21 (18)	O30—S29—C33—F35	−57.6 (2)
C28—N10—C14—C5	1.8 (3)	O31—S29—C33—F35	62.9 (2)
C12—N10—C14—C13	−1.7 (3)	O32—S29—C33—F36	62.8 (2)
C28—N10—C14—C13	−179.15 (19)	O30—S29—C33—F36	−177.05 (19)
C6—C5—C14—N10	−178.6 (2)	O31—S29—C33—F36	−56.6 (2)
C6—C5—C14—C13	2.3 (3)	O32—S29—C33—F34	−57.7 (2)
C9—C13—C14—N10	−1.8 (3)	O30—S29—C33—F34	62.4 (2)
C8—C13—C14—N10	178.23 (18)	O31—S29—C33—F34	−177.18 (18)
C9—C13—C14—C5	177.31 (18)		

### Hydrogen-bond geometry (Å, °)

**Table:**

*D*—H···*A*	*D*—H	H···*A*	*D*···*A*	*D*—H···*A*
C3—H3···O30^i^	0.93	2.57	3.449 (3)	158
C4—H4···O31^i^	0.93	2.58	3.352 (3)	141
C7—H7···O32^ii^	0.93	2.54	3.427 (3)	159
C27—H27C···O17^iii^	0.96	2.46	3.371 (3)	159
C25—H25C···Cg4^iv^	0.96	2.98	3.845 (3)	150

Symmetry codes:  (i) *x*, −*y*+3/2, *z*−1/2; (ii) *x*, −*y*+1/2, *z*−1/2; (iii) *x*, −*y*+1/2, *z*+1/2; (iv) −*x*+2, −*y*, −*z*+1.

### Table 2 S–O···π Interactions (Å,°)

**Table:**

X	*I*	*J*	*I*···*J*	X···*J*	X—*I*···*J*
S29	O31	Cg3^v^	3.968 (2)	4.111 (2)	85
S29	O32	Cg1^v^	3.178 (2)	3.757 (2)	103
S29	O32	Cg2^v^	3.512 (2)	4.741 (2)	145

Symmetry codes: (v) –*x*+1, –*y*+1, –*z*+1.Notes: *Cg*1, *Cg*2 and *Cg*3 are the centroids of the C9/N10/C11–C14, C1–C4/C11/C12 and C5–C8/C13/C14 rings, respectively.

### Table 3 π–π Interactions (Å,°)

**Table:**

*I*	*J*	*CgI*···*CgJ*	Dihedral angle	*CgI*_Perp_	*CgJ*_Perp_	*CgI*_Offset_	*CgJ*_Offset_
1	4^ii^	3.641 (2)	5.31 (10)	3.416 (2)	3.492 (2)	0.767 (2)	1.031 (2)
2	4^ii^	3.885 (2)	6.74 (11)	3.666 (2)	3.491 (2)	1.286 (2)	1.705 (2)

Symmetry code: (ii) *x*, –*y*+1/2, *z*–1/2.Notes: *Cg*1, *Cg*2 and *Cg*4 are the centroids of the C9/N10/C11–C14, C1–C4/C11/C12 and C18–C23 rings, respectively. *CgI*···*CgJ* is the distance between ring centroids. The dihedral angle is that between the planes of the rings *I* and *J*. *CgI*_Perp_ and *CgJ*_Perp_ are the perpendicular distances of *CgI* from ring *J* and of *CgJ* from ring *I*, respectively. *CgI*_Offset_ and *CgJ*_Offset_ are the distances between *CgI* and the perpendicular projection of *CgJ* on ring *I*, and between *CgJ* and the perpendicular projection of *CgI* on ring *J*, respectively.
